# Comparative efficacy and safety of antidepressant therapy for the agitation of dementia: A systematic review and network meta-analysis

**DOI:** 10.3389/fnagi.2023.1103039

**Published:** 2023-03-03

**Authors:** Kaili Chen, Haiqi Li, Le Yang, Yan Jiang, Qiaoli Wang, Jiao Zhang, Jinting He

**Affiliations:** ^1^Department of Neurology, China-Japan Union Hospital of Jilin University, Changchun, China; ^2^Department of Endocrinology, Jilin Province People's Hospital, Changchun, China

**Keywords:** dementia, agitation, antidepressant, Alzheimer's disease, network meta-analysis

## Abstract

**Background:**

Dementia is a clinical syndrome commonly seen in the elderly individuals. With the prevalence of dementia, the incidence of neuropsychiatric symptoms in dementia patients is increasing annually. Agitation, as one of the neuropsychiatric symptoms, has a serious impact on the quality of life of patients with dementia. Several antidepressant drugs have been shown to be effective for treating agitated behavior symptoms in patients with dementia, but there are no direct comparisons among those drugs. Therefore, we carried out a network meta-analysis (NMA) to examine the efficacy and safety of those antidepressant drugs.

**Methods:**

We searched eight databases (PubMed, Cochrane Library, Web of Science, Embase, Wanfang Database, China National Knowledge Infrastructure, VIP Database and China biomedical literature service) from their inception to 6 November 2022. Randomized controlled trials (RCTs) reporting the efficacy and safety of antidepressant drugs in treating agitated behavior symptoms in patients with dementia were included in our analysis. The quality assessment was carried out by two researchers individually and the analysis was based on the frequency method.

**Results:**

Twelve articles with 1,146 participants were included in our analysis. Based on the outcome of the agitation score, treatment with citalopram (standardized mean difference, SMD = −0.44, 95% confidence interval, 95% CI = −0.72 to −0.16) showed significant benefits over the placebo group. Treatment with trazodone (odds ratio, OR = 4.58, 95% CI = 1.12–18.69) was associated with a higher risk of total adverse events compared with a placebo treatment.

**Conclusion:**

Among the antidepressant drugs included in this study, treatment with citalopram was probably the only optimal intervention, when considering the improvement from baseline to the end of the intervention, and there was not a statistically significant difference in safety when compared with a placebo treatment.

**Systematic review registration:**

https://www.crd.york.ac.uk/prospero/#recordDetails, identifier: PROSPERO, CRD42022320932.

## Introduction

Dementia is a chronic, progressive syndrome that mainly manifests as memory loss, cognitive impairment, and behavioral and psychological disturbances and places a heavy challenge on human health and the global economy. The main types of dementia are Alzheimer's disease dementia, vascular dementia, frontotemporal dementia and other types of dementia (Kim and Chang, 2022). According to research statistics of the WHO Guidelines, 50 million individuals worldwide have dementia, and the population is predicted to increase to more than 150 million by 2050 (World Health Organization, 2019). Ninety percent of people with dementia suffer from one or more neuropsychiatric symptoms throughout their disease duration, including anxiety, apathy, depression, and sleep disturbances. Studies have suggested a possible correlation between neuropsychiatric symptoms and cerebrospinal fluid (CSF) biomarkers; for example, CSF tau levels may be associated with the occurrence and severity of neuropsychiatric symptoms (Cotta Ramusino et al., 2021).

A recent study indicated that more than 80% of behavioral or neuropsychiatric symptoms manifest as agitation (Cummings et al., 2015). Agitation is a group of syndromes that occurs in patients with cognitive impairment or dementia and refers to inappropriate verbal aggression, physical aggression and motor behavior that cannot be explained by a demand or by confusion of the patient's consciousness (Sano et al., 2022). Agitation impairs daily functioning, prolongs hospitalization, and is related to higher healthcare costs and death (Jones et al., 2021).

The more studied pharmacological treatments for the therapy of dementia-related agitation include antipsychotic, and antidepressant drugs, as well as cholinesterase inhibitors, meperidine and other cognition-improving-based drugs, and recently, studies have found that cannabinoids also have an effect on agitation in dementia (Ruthirakuhan et al., 2019; Magierski et al., 2020). Due to doubts about the effectiveness and safety of these medications, their use to relieve agitation is restricted. For example, the acetylcholinesterase inhibitor donepezil (Howard et al., 2007; Burns et al., 2011) and the NMDA receptor inhibitor memantine (Fox et al., 2012) were tested in randomized controlled trials but were not shown to be efficacious. Although antipsychotic drugs were used early in the treatment of agitated behavior in dementia, their efficacy was not very satisfactory (Tampi et al., 2016). A meta-analysis suggested that olanzapine and haloperidol were not significantly more effective than placebo, and risperidone was more effective but had a significant 1.7-fold increase in mortality compared to placebo (Kongpakwattana et al., 2018). Moreover, some studies have found that some antipsychotics increase the incidence of delirium and the challenge of hospitalization (Bellelli et al., 2016; Perini et al., 2019).

Antidepressant drugs are relatively safe psychotropic drugs and are commonly used in the maintenance treatment of agitated behavior in dementia (Chen et al., 2021). A meta-analysis suggested that antidepressant drugs can decrease societal costs in dementia (Huo et al., 2022). Studies have shown that some antidepressant drugs can even affect cognition in dementia. For example, studies have shown that trazodone and vortioxetine may improve cognitive function (Perini et al., 2019; Gonçalo and Vieira-Coelho, 2021). The types of antidepressant drugs in common use today are tricyclic antidepressant drugs, monoamine oxidase inhibitors, 5-hydroxytryptamine (5-HT) reuptake inhibitors, 5-HT and noradrenergic reuptake inhibitors, NE and specific serotonergic antidepressant drugs. Genetic studies suggest that the occurrence of agitated behavior in dementia may be associated with several drug targets: 5-HT receptors, serotonin transporters, and dopamine receptors, which are targets of antidepressant action (Metaxas et al., 2019; Marcinkowska et al., 2020). Several antidepressant drugs have been shown to be effective in treating dementia-related agitation symptoms of dementia (Porsteinsson et al., 2014; Zhou et al., 2019; Huang, 2021). A systematic review showed that sertraline and citalopram were efficacious in the treatment of agitated behavior in patients with dementia compared to placebo and were also better tolerated compared to typical and atypical antipsychotics (Seitz et al., 2011). Another study showed that antidepressant drugs improved neuropsychiatric symptoms, agitation, depression and the challenge of care in dementia patients with agitation (Hsu et al., 2021). All of these studies suggest the efficacy of antidepressant drug treatments for agitation symptoms in patients with dementia, but the comparative efficacy and safety among the different antidepressant drugs have not been systematically analyzed.

In view of the limitations of previous meta-analyses, we conducted a network meta-analysis to illustrate the distinctions in the efficacy and safety of different antidepressant drug treatments on agitated behavior in dementia patients and to identify the most suitable antidepressant drug treatments for the treatment of agitation in patients with dementia.

## Methods

### Study design

Our study was conducted and reported according to the Preferred Reporting Items for Systematic Reviews and Meta-analyses (PRISMA-NMA) statement (Hutton et al., 2015). Since all analyses were founded on previously published research, neither ethical review nor patient permission are necessary.

### Search strategy and selection criteria

Our search strategy and selection criteria were conducted according to the guidelines of the new edition of the Cochrane Handbook for Systematic Reviews of Interventions (Cumpston et al., 2019). We searched for relevant randomized controlled studies in several databases, from their inception dates to 6 November 2022, including PubMed, Web of Science, Cochrane Library, Embase, Wanfang Database, China National Knowledge Infrastructure, VIP and China Biomedical Literature Database. Medical subject terms and keywords were combined without consideration of publication year or language to identify relevant articles. The words below were used individually or combined with each other: “dementia,” “Alzheimer's disease,” “frontotemporal dementia,” “vascular dementia,” “agitation,” “agitated,” “antidepressant,” “tricyclic antidepressant,” “citalopram,” “escitalopram,” “mirtazapine,” “sertraline,” “fluoxetine” and “bupropion.” In addition, we also reviewed the bibliography of the included studies and previous meta-analyses of prospective studies to avoid the omission of articles for inclusion. A detailed description of the search strategies used in the database can be found in the supplementary search strategy section (Appendix 1 in Supplementary material).

### Inclusion and exclusion criteria

Articles that simultaneously satisfied all of the following requirements were eventually included in our qualitative review: (1) studies that included participants with any type of dementia diagnosed according to standardized criteria, but were not limited to meeting the standard diagnostic criteria of the NINDS-ADRDA and DSM (III-V) (McKhann et al., 1984); (2) studies that included interventions which involved any type of antidepressant (e.g., tricyclic antidepressant drugs, selective 5-HT reuptake inhibitors); (3) studies that included comparisons among various antidepressant drugs vs. a placebo or other active antidepressant drugs; (4) trials that were RCTs only; (5) studies that included improvements in agitation scores compared with baseline as the outcome measure for efficacy, which was mainly measured by the Cohen-Mansfield Agitation Inventory (CMAI); if not available, then the agitation subscale of NBRS, the agitation subscale of the NPI, or the Agitated Behavior in Dementia Scale could also be used to assess agitation; and (6) studies that included the incidence of total adverse events (AEs) during treatment as the outcome measure for safety. Studies that had any of the following components were excluded: (1) the studies compared antidepressant drugs with non-antidepressant drugs (e.g., antipsychotics, cannabinoids); or (2) the studies were case-control studies, open-label trials, protocols, reviews, or meta-analyses.

### Literature selection

All articles searched from the eight databases were imported into Endnote X20 reference management software (Thompson ISI Research Soft, Philadelphia, PA) for screening, and any duplicate or overlapping publications were excluded. The selection of the literature was carried out independently by two researchers, and any differences of opinion could be discussed and resolved, if necessary, by consultation with a third researcher.

### Quality assessment

Two researchers individually evaluated the quality of the included studies by using RevMan 5.4 software, according to the Cochrane Collaboration's tool for assessing risk of bias in randomized trials (Higgins et al., 2011), and consensus was reached through debate if there was a disagreement. If there was still disagreement, a third researcher conducted a further assessment.

### Data extraction

Two authors independently collected the relevant information according to the data extraction strategy that followed the Cochrane Consumer and Communications Review Group and included the following: (1) publication information: title, year of publication and last name of the first author; (2) research subjects: type of dementia and demographic data such as sex ratio, age and the number of participants; (3) intervention details: dose and treatment duration; (4) outcome measures of efficacy: baseline and endpoint information for intervention and control groups, mainly including total numbers, means and standard deviations; and (5) outcome measures of safety: baseline and endpoint information for intervention and control groups, mainly including the total and adverse reaction numbers of participants.

### Statistical analyses

We analyzed the effectiveness and safety of all the interventions in all of the included studies based on the frequency-based theory of network meta-analysis. Outcome indicators for efficacy were continuous variables, and SMD with 95% CIs were used to show the effect size; outcome indicators for safety were categorical variables, and OR with 95% CIs were used to show the effect size. Comparisons of the different interventions are shown in the network evidence map plots, where the dimension of the nodes represents the number of study participants and the breadth of the connecting line represents the number of studies for each treatment. We did not conduct inconsistency tests due to the inclusion of articles that were all comparisons of antidepressant drugs vs. placebo and the lack of direct comparisons between antidepressant drugs. When the included studies used different doses or durations for the same intervention, we combined them into one result. The area under the surface of the cumulative ranking curve (SUCRA) was used to express the efficacy and safety ranking of the various treatments. We used Stata 14.2 (Stata, Corp, LP College Station, TX) for statistical analysis of the data.

## Results

### Literature search and selection

**Figure 1:**
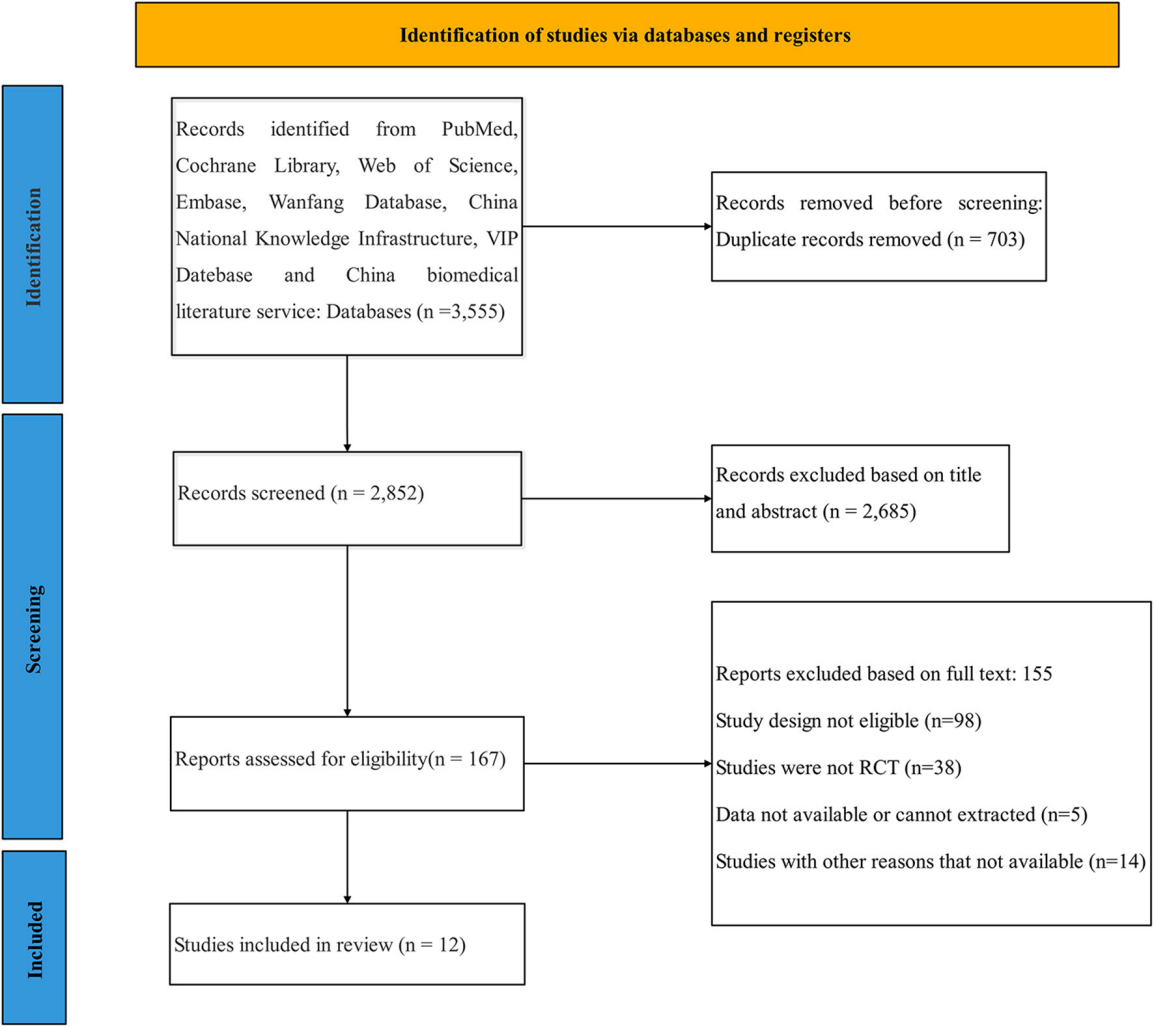
Literature review flowchart.

We obtained a total of 3,555 articles according to the predetermined search strategy. We removed 703 duplicates that were identified by the reference management software, and then excluded 2,685 articles by reading the title and abstract and excluded 155 articles according to the inclusion criteria and data integrity by reading the full text. Although the data on the efficacy of escitalopram and sertraline were incomplete, we included those analyses because of the detailed data regarding their safety (Lanctot et al., 2002; Huang, 2021). However, one study about trazodone was excluded because of the lack of baseline data (Weiner et al., 2002). Finally, a total of 12 studies were included. The flow chart of the screening process is provided in Figure 1.

### Baseline characteristics and quality assessment

In our study, we included four studies on citalopram, three studies on sertraline, two studies on trazodone, one study on fluoxetine, one study on mirtazapine and one study on escitalopram. The included studies involved 1,146 individuals. The percentage of female participants ranged between 40.54% and 100%. The average age of the patients ranged between 61.7 and 90.1 years old. The duration of these studies ranged between 17 days and 16 weeks. The primary characteristics of the included studies are shown in Table 1. Each study was assessed qualitatively by using the Cochrane tool for various indicators. Overall, most of the randomized controlled trials generated random sequences with low risks of bias. The risk of bias assessment for the included studies is presented in Appendix 2 in Supplementary material. Publication bias was presented using a funnel plot (Appendix 3 in Supplementary material), and it showed that there may be no publication bias in this study.

**Table 1:** The characteristics and demographics of the included studies.

	**References**	**Participants**	**Number**	**Therapy**	**Sex (female%)**	**Age (years)**	**Study duration**
1	Banerjee et al. (2021)	AD	102	Mirtazapine (45 mg/d)	74.51	82.2	12 weeks
			102	Placebo	57.84	82.8	
2	Huang (2021)	AD	50	Escitalopram (10–30 mg/d)	50.00	69.78	90 days
			50	Placebo	50.00	69.81	
3	Zhou et al. (2019)	AD	40	Citalopram (10–30 mg/d)	60.0	71.00	12 weeks
			40	Placebo	57.5	71.10	
4	Porsteinsson et al. (2014)	AD	94	Citalopram (30 mg/d)	53.19	78.00	9 weeks
			92	Placebo	55.43	79.00	
5	Lebert et al. (2004)	FTD	15	Trazodone (50–300 mg/d)	51.61	61.70	6 weeks
			16	Placebo			
6	Finkel et al. (2004)	AD	124	Sertraline (50–200 mg/d)	61.29	75.70	12 weeks
			120	Placebo	56.67	76.90	
7	Lanctot et al. (2002)	AD	22	Sertraline (50–100 mg/d)	47.62	82.00	6 weeks
			22	Placebo			
8	Pollock et al. (2002)	AD	31	Citalopram (10–20 mg/d)	61.30	80.9	17 days
			21	Placebo	57.14	78.5	
9	Magai et al. (2000)	AD	17	Sertraline (100 mg/d)	100	88.40	8 weeks
			14	Placebo		90.10	
10	Teri et al. (2000)	AD	37	Trazodone (50 mg/d)	40.54	73.2	16 weeks
			36	Placebo	66.67	75.8	
11	Auchus and Bissey-Black (1997)	AD	6	Fluoxetine (20 mg/d)	67.0	75.6	9 weeks
			6	Placebo			
12	Nyth and Gottfries (1990)	AD, VD	44	Citalopram (10–30 mg/d)	77.53	77.6	4 weeks
			45	Placebo			

AD, Alzheimer's disease; VD, vascular dementia; FTD, frontotemporal dementia.

### Efficacy comparison

**Figure 2:**
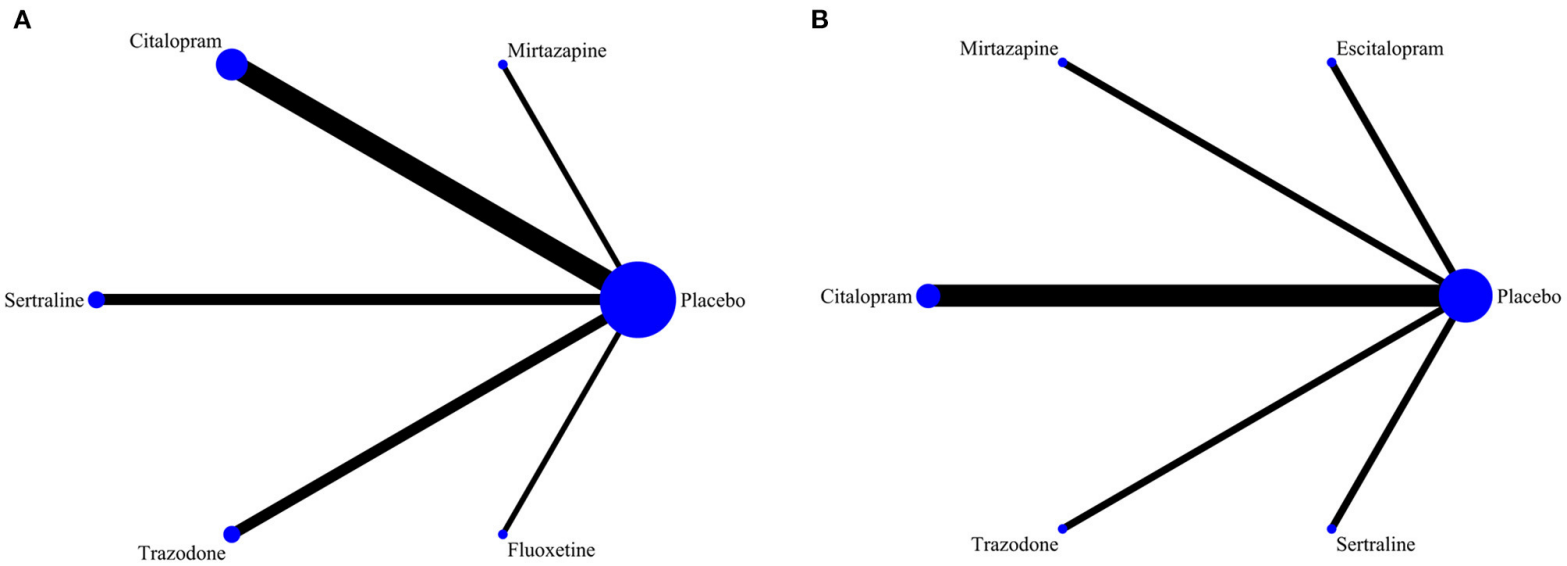
**(A)** Network diagram of the efficacy of different drug therapies for agitation symptoms in patients with dementia. **(B)** Network diagram of the safety of different drug therapies for agitation symptoms in patients with dementia.

Among the 10 included RCTs, 5 kinds of antidepressant drugs were tested. The network diagram of antidepressant drug efficacy is shown in Figure 2A. All RCTs were comparisons between antidepressant drugs and a placebo, and there were no direct comparisons between different antidepressant drugs, so we did not need to examine inconsistencies between direct and indirect treatments. At the same time, the efficacy of citalopram (SMD = −0.44, 95% CI −0.72 to −0.16) was significantly higher than that of a placebo. Furthermore, we observed non-significant effects of sertraline (SMD = −0.08, 95% CI −0.43 to 0.27), mirtazapine (SMD = −0.04, 95% CI −0.45 to 0.37), trazodone (SMD = 0.03, 95% CI −0.43 to 0.49) and fluoxetine (SMD = 0.31, 95% CI −0.87 to 1.49) compared with a placebo. A league table for antidepressant drug efficacy is shown in Table 2A. The ranking of antidepressant drug efficacy was achieved with the SUCRA line-rank, which indicated that citalopram had the highest probability (SUCRA 94.8%) compared with the other antidepressant drugs, followed by sertraline (53.9%), mirtazapine (46.8%), placebo (40.3%), trazodone (38.2%) and fluoxetine (26.1%).

**Table 2A:** League table for antidepressant drug efficacy.

Citalopram	0.36	0.40	0.44	0.47	0.75
	−0.09 to 0.80	−0.09 to 0.90	0.16 to 0.72	−0.05 to 0.99	−0.46 to 1.96
−0.36	Sertraline	0.05	0.08	0.11	0.39
−0.80 to 0.09		−0.49 to 0.59	−0.27 to 0.43	−0.46 to 0.69	−0.84 to 1.63
−0.40	−0.05	Mirtazapine	0.04	0.07	0.35
−0.90 to 0.09	−0.59 to 0.49		−0.37 to 0.45	−0.55 to 0.68	−0.90 to 1.60
−0.44	−0.08	−0.04	Placebo	0.03	0.31
−0.72 to −0.16	−0.43 to 0.27	−0.45 to 0.37		−0.43 to 0.49	−0.87 to 1.49
−0.47	−0.11	−0.07	−0.03	Trazodone	0.28
−0.99 to 0.05	−0.69 to 0.46	−0.68 to 0.55	−0.49 to 0.43		−0.99 to 1.55
−0.75	−0.39	−0.35	−0.31	−0.28	Fluoxetine
−1.96 to 0.46	−1.63 to 0.84	−1.60 to 0.90	−1.49 to 0.87	−1.55 to 0.99	

Words in blue boxes are different antidepressant drugs.

### Safety comparison

Detailed data for total AEs were reported in 7 RCTs in our study. The network diagram of the safety of the different antidepressant drugs is represented in Figure 2B. Among the many adverse effects, the most frequently reported effects were dizziness, tremors, and weakness. Our study showed that the incidence of total AEs reported for trazodone (OR = 4.58, 95% CI = 1.12–18.69) was significantly higher than that reported for placebo. A league table for antidepressant drug safety is shown in Table 2B. The probability of AEs associated with each antidepressant drug in the analysis, in order from highest to lowest, was as follows: trazodone (96.5%), sertraline (51.7%), mirtazapine (48.8%), citalopram (41.3%), placebo (40.3%) and escitalopram (21.2%).

**Table 2B:** League table for antidepressant drug safety.

Trazodone	0.26	0.24	0.22	0.22	0.16
	0.04–1.65	0.05–1.09	0.05–1.05	0.05–0.89	0.03–0.85
3.82	Sertraline	0.91	0.84	0.83	0.61
0.61–24.00		0.24–3.39	0.22–3.28	0.25–2.72	0.14–2.70
4.21	1.10	Mirtazapine	0.93	0.92	0.68
0.92–19.20	0.30–4.11		0.38–2.24	0.52–1.63	0.23–1.95
4.54	1.19	1.08	Citalopram	0.99	0.73
0.96–21.59	0.30–4.65	0.45–2.62		0.51–1.94	0.24–2.23
4.58	1.20	1.09	1.01	Placebo	0.74
1.12–18.69	0.37–3.92	0.61–1.94	0.51–1.98		0.30–1.79
6.22	1.63	1.48	1.37	1.36	Escitalopram
1.18–32.78	0.37–7.15	0.51–4.26	0.45–4.17	0.56–3.30	

Words in blue boxes are different antidepressant drugs.

## Discussion

With the advent of population aging all over the world, the incidence of dementia is increasing year by year, as is the prevalence of its associated neuropsychiatric symptoms, especially agitation; therefore, there is an urgent need to find effective and safe drugs. A randomized controlled trial of citalopram in the treatment of agitation in dementia yielded significant results by Nyth and Gottfries (1990). Recently, with the development of massive clinical trials, antidepressant drugs have become a more effective and safer drug for treating agitation in dementia patients. The antidepressant drugs that are used for the treatment of agitated behavior in dementia include citalopram, mirtazapine, sertraline, trazodone, fluoxetine, escitalopram and others. Although there is evidence to support antidepressant drug use for the treatment of agitation in patients with dementia, comparisons of the efficacy and safety of different antidepressant drug treatments have never been made. According to the statistical results, we concluded that citalopram was the only significantly effective and relatively safe antidepressant to treat agitation in patients with dementia at this time.

The results of our study revealed that the use of citalopram, a 5-HT antidepressant is efficacious for treating agitation symptoms in patients with dementia, which is consistent with the findings of another study published in 2021 which revealed the efficacy of 5-HT antidepressant drug use for the treatment of agitation symptoms (Hsu et al., 2021). In addition, studies have shown that a reduction in 5-HT1AR in the cerebral cortex is directly related to the occurrence of agitation in AD patients (Lai et al., 2003). A recent study showed that treatment with citalopram significantly reduced agitated behavior symptoms in people with dementia and had fewer adverse effects than antipsychotic drugs such as risperidone, quetiapine and olanzapine (Qasim and Simpson, 2022). The results of our study also revealed that treatment with other antidepressant drugs such as mirtazapine, sertraline, and trazodone is not significantly in efficacious when compared to a placebo, although some studies have shown their efficacy in treating agitation (Pollock et al., 2002; Seitz et al., 2011). More randomized controlled trials should be conducted to verify those results.

In the safety analysis, we only analyzed articles that did report adverse reactions. Among the articles we included, the main common adverse reactions were dizziness, headache, nausea, weakness or fatigue, and diarrhea. We selected total adverse reactions as the outcome indicator of safety because it was important factor that could have led to a large number of participants withdrawing. Regarding total AEs, escitalopram, mirtazapine, citalopram and sertraline treatments were not significantly different from a placebo. Because the total AEs associated with trazodone use were significantly higher than those associated with taking a placebo, we consider that trazodone is probably not an appropriate antidepressant drug for the treatment of agitation symptoms in patients with dementia.

Our study demonstrates the uniqueness of the antidepressant drug citalopram for treating agitated behavior symptoms in patients with dementia. However, it has been noted that citalopram can extend negative side effects such as the QT interval. The results of the CitAD trial revealed that 30 mg of citalopram daily improved BPSD symptoms and agitation scores in non-depressed patients with Alzheimer's disease. However, that study also found that the risk of QT interval prolongation may be higher in the citalopram 30 mg treatment group than in the placebo group; that side effect may be dose-related, which raises concerns about the use of citalopram (Porsteinsson et al., 2014). However, two sizable population-based cohort investigations did not show an elevated incidence of ventricular arrhythmias or other mortality, thus casting doubt on the citalopram risk-related concerns (Zivin et al., 2013; Ray et al., 2017). Studies have confirmed that QT prolongation with citalopram treatment or the use of other antidepressant drugs is modest and does not exacerbate increased mortality due to cardiac risk (Castro et al., 2013).

A subsequent analysis showed that treatment with R-citalopram was associated with adverse effects while S-citalopram was associated with better efficacy (Ho et al., 2016), which provided ideas for the treatment of agitation in patients with dementia. Therefore, attention has been directed to escitalopram, which is S-citalopram. A study demonstrated that escitalopram has a good effect on agitated behavior symptoms in Alzheimer's disease patients, but detailed data on efficacy are not available (Huang, 2021). Our study demonstrated that treatment with escitalopram was not significantly different from treatment with a placebo regarding the total AEs, and even had a lower sura score ranking than that of the placebo, probably because the number of participants in this study was small. A recent NIH-funded, randomized multicentre clinical trial, S-CitAD (Ehrhardt et al., 2019), was aimed at investigating the effects of escitalopram treatment on agitated behavior symptoms in patients with Alzheimer's disease by giving patients 15 mg of escitalopram daily or an identically packaged placebo; the results on the efficacy of escitalopram treatment for agitation symptoms in patients with dementia are believed to be forthcoming.

## Strengths and limitations

In our study, we analyzed all the antidepressant drugs used to treat agitated behavior symptoms in patients with dementia and found that only citalopram was effective and relatively safe. The studies included in our analysis investigated antidepressant drugs for the treatment of agitation in various populations of patients with different types of dementia. The included studies provided evidence for antidepressant drug selection for treating agitation symptoms in dementia patients. However, there are some limitations. First, we did not perform subgroup analyses because the number of included studies was small. There were many different factors among the included studies, such as the types of dementia, the doses of the antidepressant drugs, the durations of the treatments and the types of agitation symptom scales. Second, we assessed the safety of the treatment. Antidepressant drugs with similar safety results compared with placebo were not always safe or without adverse events. Third, regarding the availability of literature, we included a relatively small number of articles among the different groups, and some articles were not included in the efficacy or safety analysis because of limited data. Hence, higher-quality and large-sample studies are needed to verify our results.

## Conclusion

Citalopram was the only effective antidepressant drug for the treatment of agitation symptoms in patients with dementia according to our NMA results. However, given the limited number and small sample size of available RCTs, other potential risks of bias, and some variation in study design, such as different doses and durations of interventions, higher-quality RCTs with large sample sizes are needed to confirm our results in the future.

## Data availability statement

The original contributions presented in the study are included in the article/Supplementary material, further inquiries can be directed to the corresponding author.

## Author contributions

KC, HL, LY, YJ, and JH served as the principal authors and designed the study. JH contributed to conceptualization, methodology, supervision, funding acquisition, project administration, and revised the article. YJ, QW, and JZ contributed to the screening of the literature and the data extraction. KC, HL, and LY wrote the manuscript. All authors contributed to the article and approved the submitted version.
