# Supplementary material for: Comparative efficacy and safety of antidepressant therapy for the agitation of dementia: A systematic review and network meta-analysis
Source: Front Aging Neurosci. 2023 Mar 3;15:1103039. doi: 10.3389/fnagi.2023.1103039 (PMC10020338; doi:10.3389/fnagi.2023.1103039)
Supplement: Supplementary file 1 [file Data_Sheet_1.docx]

**Pubmed Search strategy; searched on November 6, 2022**

#1 Search “Dementia”[Mesh]

#2 Search (Dementia [Title/Abstract]) OR (Dementias [Title/Abstract]) OR (Amentia [Title/Abstract]) OR (Amentias [Title/Abstract]) OR (Senile Paranoid Dementia [Title/Abstract]) OR (Dementias, Senile Paranoid [Title/Abstract]) OR (Paranoid Dementia, Senile [Title/Abstract]) OR (Paranoid Dementias, Senile [Title/Abstract]) OR (Senile Paranoid Dementias [Title/Abstract]) OR (Familial Dementia [Title/Abstract]) OR (Dementia, Familial [Title/Abstract]) OR (Dementias, Familial [Title/Abstract]) OR (Familial Dementias [Title/Abstract]) OR (Demented [Title/Abstract])

#3 Search (“Alzheimer Disease”[Mesh])

#4 Search (Alzheimer Disease [Title/Abstract]) OR (Alzheimer Dementia [Title/Abstract]) OR (Alzheimer Dementias [Title/Abstract]) OR (Dementia, Alzheimer [Title/Abstract]) OR (Alzheimer's Disease [Title/Abstract]) OR (Dementia, Senile [Title/Abstract]) OR (Senile Dementia [Title/Abstract]) OR (Dementia, Alzheimer Type [Title/Abstract]) OR (Alzheimer Type Dementia [Title/Abstract]) OR (Alzheimer-Type Dementia (ATD) [Title/Abstract]) OR (Alzheimer Type Dementia (ATD) [Title/Abstract]) OR (Dementia, Alzheimer-Type (ATD) [Title/Abstract]) OR (Alzheimer Type Senile Dementia [Title/Abstract]) OR (Primary Senile Degenerative Dementia [Title/Abstract]) OR (Dementia, Primary Senile Degenerative [Title/Abstract]) OR (Alzheimer Sclerosis [Title/Abstract]) OR (Sclerosis, Alzheimer [Title/Abstract]) OR (Alzheimer Syndrome [Title/Abstract]) OR (Alzheimer's Diseases [Title/Abstract]) OR (Alzheimer Diseases [Title/Abstract]) OR (Alzheimers Diseases [Title/Abstract]) OR (Senile Dementia, Alzheimer Type [Title/Abstract]) OR (Acute Confusional Senile Dementia [Title/Abstract]) OR (Senile Dementia, Acute Confusional [Title/Abstract]) OR (Dementia, Presenile [Title/Abstract]) OR (Presenile Dementia [Title/Abstract]) OR (Alzheimer Disease, Late Onset [Title/Abstract]) OR (Late Onset Alzheimer Disease [Title/Abstract]) OR (Alzheimer's Disease, Focal Onset [Title/Abstract]) OR (Focal Onset Alzheimer's Disease [Title/Abstract]) OR (Familial Alzheimer Disease (FAD) [Title/Abstract]) OR (Alzheimer Disease, Familial (FAD) [Title/Abstract]) OR (Familial Alzheimer Diseases (FAD) [Title/Abstract]) OR (Alzheimer Disease, Early Onset [Title/Abstract]) OR (Early Onset Alzheimer Disease [Title/Abstract]) OR (Presenile Alzheimer Dementia [Title/Abstract]) OR (Alzheimer [Title/Abstract])

#5 Search “Frontotemporal Dementia”[Mesh]

#6 Search (Frontotemporal Dementia [Title/Abstract]) OR (Dementias, Frontotemporal [Title/Abstract]) OR (Frontotemporal Dementias [Title/Abstract]) OR (Frontotemporal Dementia with Parkinsonism [Title/Abstract]) OR (Dementia, Frontotemporal, with Parkinsonism [Title/Abstract]) OR (Multiple System Tauopathy with Presenile Dementia [Title/Abstract]) OR (Frontotemporal Dementia, Ubiquitin-Positive [Title/Abstract]) OR (Dementia, Ubiquitin-Positive Frontotemporal [Title/Abstract]) OR (Dementias, Ubiquitin-Positive Frontotemporal [Title/Abstract]) OR (Frontotemporal Dementia, Ubiquitin Positive [Title/Abstract]) OR (Frontotemporal Dementias, Ubiquitin-Positive [Title/Abstract]) OR (Ubiquitin-Positive Frontotemporal Dementia [Title/Abstract]) OR (Ubiquitin-Positive Frontotemporal Dementias [Title/Abstract]) OR (Dementia, Hereditary Dysphasic Disinhibition [Title/Abstract]) OR (Dementia, Frontotemporal [Title/Abstract]) OR (Frontotemporal Lobe Dementia [Title/Abstract]) OR (Dementia, Frontotemporal Lobe [Title/Abstract]) OR (Dementias, Frontotemporal Lobe [Title/Abstract]) OR (Frontotemporal Lobe Dementias [Title/Abstract]) OR (Lobe Dementia, Frontotemporal [Title/Abstract]) OR (Lobe Dementias, Frontotemporal [Title/Abstract]) OR (Wilhelmsen-Lynch Disease [Title/Abstract]) OR (Disease, Wilhelmsen-Lynch [Title/Abstract]) OR (Diseases, Wilhelmsen-Lynch [Title/Abstract]) OR (Wilhelmsen Lynch Disease [Title/Abstract]) OR (Wilhelmsen-Lynch Diseases [Title/Abstract]) OR (Frontotemporal Lobar Degeneration With Ubiquitin-Positive Inclusions [Title/Abstract]) OR (Frontotemporal Lobar Degeneration With Ubiquitin Positive Inclusions [Title/Abstract]) OR (Disinhibition-Dementia-Parkinsonism-Amyotrophy Complex [Title/Abstract]) OR (Complex, Disinhibition-Dementia-Parkinsonism-Amyotrophy [Title/Abstract]) OR (Complices, Disinhibition-Dementia-Parkinsonism-Amyotrophy [Title/Abstract]) OR (Disinhibition Dementia Parkinsonism Amyotrophy Complex [Title/Abstract]) OR (Disinhibition-Dementia-Parkinsonism-Amyotrophy Complices [Title/Abstract]) OR (Frontotemporal Dementia with Parkinsonism-17 [Title/Abstract]) OR (Frontotemporal Dementia with Parkinsonism 17 [Title/Abstract]) OR (Disinhibition-Dementia-Parkinsonism-Amytrophy Complex [Title/Abstract]) OR (Complex, Disinhibition-Dementia-Parkinsonism-Amytrophy [Title/Abstract]) OR (Complices, Disinhibition-Dementia-Parkinsonism-Amytrophy [Title/Abstract]) OR (Disinhibition Dementia Parkinsonism Amytrophy Complex [Title/Abstract]) OR (Disinhibition-Dementia-Parkinsonism-Amytrophy Complices [Title/Abstract]) OR (Familial Pick's Disease [Title/Abstract]) OR (Disease, Familial Pick's [Title/Abstract]) OR (Diseases, Familial Pick's [Title/Abstract]) OR (Familial Pick Disease [Title/Abstract]) OR (Familial Pick's Diseases [Title/Abstract]) OR (Familial Picks Disease [Title/Abstract]) OR (Pick's Disease, Familial [Title/Abstract]) OR (Pick's Diseases, Familial [Title/Abstract]) OR (Hereditary Dysphasic Disinhibition Dementia [Title/Abstract]) OR (FTDP-17 [Title/Abstract]) OR (DDPAC [Title/Abstract]) OR (GRN-Related Frontotemporal Dementia [Title/Abstract]) OR (Dementia, GRN-Related Frontotemporal [Title/Abstract]) OR (Dementias, GRN-Related Frontotemporal [Title/Abstract]) OR (Frontotemporal Dementia, GRN-Related [Title/Abstract]) OR (Frontotemporal Dementias, GRN-Related [Title/Abstract]) OR (GRN Related Frontotemporal Dementia [Title/Abstract]) OR (GRN-Related Frontotemporal Dementias [Title/Abstract]) OR (FTD-GRN [Title/Abstract]) OR (FTD-PGRN [Title/Abstract]) OR (FTLD-17 GRN [Title/Abstract]) OR (FTLD with TDP-43 Pathology [Title/Abstract]) OR (FTLD with TDP 43 Pathology [Title/Abstract]) OR (FTLD-TDP [Title/Abstract]) OR (HDDD1 [Title/Abstract]) OR (HDDD2 [Title/Abstract]) OR (Frontotemporal Lobe Dementia (FLDEM) [Title/Abstract]) OR (Dementia, Frontotemporal Lobe (FLDEM) [Title/Abstract]) OR (Dementias, Frontotemporal Lobe (FLDEM) [Title/Abstract]) OR (Frontotemporal Lobe Dementias (FLDEM) [Title/Abstract]) OR (Semantic Dementia [Title/Abstract]) OR (Dementia, Semantic [Title/Abstract]) OR (Dementias, Semantic [Title/Abstract]) OR (Semantic Dementias [Title/Abstract])

#7 Search “Dementia, Vascular”[Mesh]

#8 Search (Dementia, Vascular [Title/Abstract]) OR (Dementias, Vascular [Title/Abstract]) OR (Vascular Dementias [Title/Abstract]) OR (Vascular Dementia [Title/Abstract]) OR (Vascular Dementia, Acute Onset [Title/Abstract]) OR (Acute Onset Vascular Dementia [Title/Abstract]) OR (Subcortical Vascular Dementia [Title/Abstract]) OR (Dementia, Subcortical Vascular [Title/Abstract]) OR (Dementias, Subcortical Vascular [Title/Abstract]) OR (Subcortical Vascular Dementias [Title/Abstract]) OR (Vascular Dementia, Subcortical [Title/Abstract]) OR (Vascular Dementias, Subcortical [Title/Abstract]) OR (Arteriosclerotic Dementia [Title/Abstract]) OR (Arteriosclerotic Dementias [Title/Abstract]) OR (Dementia, Arteriosclerotic [Title/Abstract]) OR (Dementias, Arteriosclerotic [Title/Abstract]) OR (Binswanger Disease [Title/Abstract]) OR (Disease, Binswanger [Title/Abstract]) OR (Chronic Progressive Subcortical Encephalopathy [Title/Abstract]) OR (Binswanger Encephalopathy [Title/Abstract]) OR (Leukoencephalopathy, Subcortical [Title/Abstract]) OR (Leukoencephalopathies, Subcortical [Title/Abstract]) OR (Subcortical Leukoencephalopathies [Title/Abstract]) OR (Encephalopathy, Subcortical Arteriosclerotic [Title/Abstract]) OR (Binswanger's Disease [Title/Abstract]) OR (Binswangers Disease [Title/Abstract]) OR (Disease, Binswanger's [Title/Abstract]) OR (Encephalopathy, Subcortical, Chronic Progressive [Title/Abstract]) OR (Subcortical Encephalopathy, Chronic Progressive [Title/Abstract]) OR (Subcortical Leukoencephalopathy [Title/Abstract]) OR (Subcortical Arteriosclerotic Encephalopathy [Title/Abstract]) OR (Arteriosclerotic Encephalopathy, Subcortical [Title/Abstract]) OR (Arteriosclerotic Encephalopathies, Subcortical [Title/Abstract]) OR (Encephalopathies, Subcortical Arteriosclerotic [Title/Abstract]) OR (Subcortical Arteriosclerotic Encephalopathies [Title/Abstract]) OR (Encephalopathy, Binswanger's [Title/Abstract]) OR (Binswanger's Encephalopathy [Title/Abstract]) OR (Encephalopathy, Binswangers [Title/Abstract]) OR (Encephalopathy, Binswanger [Title/Abstract]) OR (Encephalopathy, Chronic Progressive Subcortical [Title/Abstract])

#9 Search “Lewy body, dementia”[Mesh]

#10 Search (Lewy body, dementia [Title/Abstract]) OR (Diffuse Lewy Body Disease [Title/Abstract]) OR (Lewy Body Dementia [Title/Abstract]) OR (Cortical Lewy Body Disease [Title/Abstract]) OR (Lewy Body Disease, Cortical [Title/Abstract]) OR (Lewy Body Type Senile Dementia [Title/Abstract]) OR (Lewy Body Disease, Diffuse [Title/Abstract]) OR (Dementia, Lewy Body [Title/Abstract])

#11 Search “Psychomotor Agitation”[Mesh]

#12 Search (Psychomotor Agitation [Title/Abstract]) OR (Agitation [Title/Abstract]) OR (Restlessness [Title/Abstract]) OR (Psychomotor Hyperactivity [Title/Abstract]) OR (Hyperactivity, Psychomotor [Title/Abstract]) OR (Psychomotor Restlessness [Title/Abstract]) OR (Restlessness, Psychomotor [Title/Abstract]) OR (Agitation, Psychomotor [Title/Abstract]) OR (Excitement, Psychomotor [Title/Abstract]) OR (Psychomotor Excitement [Title/Abstract]) OR (Akathisia [Title/Abstract]) OR (agitated [Title/Abstract]) OR (Aggressive [Title/Abstract])

#13 Search “Antidepressive Agents”[Mesh]

#14 Search (Antidepressive Agents [Title/Abstract]) OR (Antidepressant Drug [Title/Abstract]) OR (Drug, Antidepressant [Title/Abstract]) OR (Antidepressants [Title/Abstract]) OR (Antidepressant [Title/Abstract]) OR (Antidepressant Drugs [Title/Abstract]) OR (Antidepressant Medication [Title/Abstract]) OR (Medication, Antidepressant [Title/Abstract]) OR (Antidepressive Agent [Title/Abstract]) OR (Agent, Antidepressive [Title/Abstract]) OR (Thymoanaleptics [Title/Abstract]) OR (Thymoanaleptic [Title/Abstract]) OR (Thymoleptics [Title/Abstract]) OR (Thymoleptic [Title/Abstract]) OR (antidepressant agent [Title/Abstract])

#15 Search “Serotonin Uptake Inhibitors”[Mesh]

#16 Search (Serotonin Uptake Inhibitors [Title/Abstract]) OR (Reuptake Inhibitors, Serotonin [Title/Abstract]) OR (Uptake Inhibitors, 5-Hydroxytryptamine [Title/Abstract]) OR (Uptake Inhibitors, 5 Hydroxytryptamine [Title/Abstract]) OR (Uptake Inhibitors, Serotonin [Title/Abstract]) OR (Serotonin Reuptake Inhibitor [Title/Abstract]) OR (Inhibitor, Serotonin Reuptake [Title/Abstract]) OR (Reuptake Inhibitor, Serotonin [Title/Abstract]) OR (Serotonin Uptake Inhibitor [Title/Abstract]) OR (Inhibitor, Serotonin Uptake [Title/Abstract]) OR (Uptake Inhibitor, Serotonin [Title/Abstract]) OR (5-HT Uptake Inhibitor [Title/Abstract]) OR (5 HT Uptake Inhibitor [Title/Abstract]) OR (Inhibitor, 5-HT Uptake [Title/Abstract]) OR (Uptake Inhibitor, 5-HT [Title/Abstract]) OR (5-Hydroxytryptamine Uptake Inhibitor [Title/Abstract]) OR (5 Hydroxytryptamine Uptake Inhibitor [Title/Abstract]) OR (Inhibitor, 5-Hydroxytryptamine Uptake [Title/Abstract]) OR (Uptake Inhibitor, 5-Hydroxytryptamine [Title/Abstract]) OR (5-HT Uptake Inhibitors [Title/Abstract]) OR (5 HT Uptake Inhibitors [Title/Abstract]) OR (5-Hydroxytryptamine Uptake Inhibitors [Title/Abstract]) OR (5 Hydroxytryptamine Uptake Inhibitors [Title/Abstract]) OR (Inhibitors, 5-HT Uptake [Title/Abstract]) OR (Inhibitors, 5 HT Uptake [Title/Abstract]) OR (Inhibitors, 5-Hydroxytryptamine Uptake [Title/Abstract]) OR (Inhibitors, 5 Hydroxytryptamine Uptake [Title/Abstract]) OR (Inhibitors, Serotonin Reuptake [Title/Abstract]) OR (Inhibitors, Serotonin Uptake [Title/Abstract]) OR (Serotonin Reuptake Inhibitors [Title/Abstract]) OR (Uptake Inhibitors, 5-HT [Title/Abstract]) OR (Uptake Inhibitors, 5 HT [Title/Abstract]) OR (Selective Serotonin Reuptake Inhibitors [Title/Abstract]) OR (Selective Serotonin Reuptake Inhibitor [Title/Abstract])

#17 Search “Antidepressive Agents, Tricyclic”[Mesh]

#18 Search (Antidepressive Agents, Tricyclic [Title/Abstract]) OR (Agents, Tricyclic Antidepressive [Title/Abstract]) OR (Tricyclic Antidepressant Drug [Title/Abstract]) OR (Antidepressant Drug, Tricyclic [Title/Abstract]) OR (Drug, Tricyclic Antidepressant [Title/Abstract]) OR (Tricyclic Antidepressant [Title/Abstract]) OR (Antidepressant, Tricyclic [Title/Abstract]) OR (Antidepressants, Tricyclic [Title/Abstract]) OR (Tricyclic Antidepressants [Title/Abstract]) OR (Tricyclic Antidepressive Agents [Title/Abstract]) OR (Tricyclic Antidepressive Agent [Title/Abstract]) OR (Agent, Tricyclic Antidepressive [Title/Abstract]) OR (Antidepressive Agent, Tricyclic [Title/Abstract]) OR (Antidepressant Drugs, Tricyclic [Title/Abstract]) OR (Drugs, Tricyclic Antidepressant [Title/Abstract]) OR (Tricyclic Antidepressant Drugs [Title/Abstract])

#19 Search “Monoamine Oxidase Inhibitors”[Mesh]

#20 Search (Monoamine Oxidase Inhibitors [Title/Abstract]) OR (Inhibitors, Monoamine Oxidase [Title/Abstract]) OR (Monoamine Oxidase Inhibitor [Title/Abstract]) OR (Inhibitor, Monoamine Oxidase [Title/Abstract]) OR (MAO Inhibitors [Title/Abstract]) OR (Inhibitors, MAO [Title/Abstract]) OR (MAO Inhibitor [Title/Abstract]) OR (Inhibitor, MAO [Title/Abstract]) OR (RIMA (Reversible Inhibitor of Monoamine Oxidase A) [Title/Abstract]) OR (Reversible Inhibitors of Monoamine Oxidase [Title/Abstract]) OR (Reversible Inhibitor of Monoamine Oxidase [Title/Abstract])

#21 Search “Citalopram”[Mesh]

#22 Search (Citalopram [Title/Abstract]) OR (Cytalopram [Title/Abstract]) OR (Citalopram Hydrobromide [Title/Abstract]) OR (Lu-10-171 [Title/Abstract]) OR (Lu10171 [Title/Abstract]) OR (Seropram [Title/Abstract]) OR (Celexa [Title/Abstract])

#23 Search “Escitalopram”[Mesh]

#24 Search (Escitalopram [Title/Abstract]) OR (Escitalopram Oxalate [Title/Abstract]) OR (Lexapro [Title/Abstract])

#25 Search “Paroxetine”[Mesh]

#26 Search (Paroxetine [Title/Abstract]) OR (Aropax [Title/Abstract]) OR (BRL-29060 [Title/Abstract]) OR (BRL 29060 [Title/Abstract]) OR (BRL29060 [Title/Abstract]) OR (FG-7051 [Title/Abstract]) OR (FG 7051 [Title/Abstract]) OR (FG7051 [Title/Abstract]) OR (Paroxetine Acetate [Title/Abstract]) OR (Seroxat [Title/Abstract]) OR (Paroxetine Hydrochloride Anhydrous [Title/Abstract]) OR (Paroxetine Maleate [Title/Abstract]) OR (Paroxetine, cis-(+)-Isomer [Title/Abstract]) OR (Paroxetine, cis-(-)-Isomer [Title/Abstract]) OR (Paroxetine, trans-(+)-Isomer [Title/Abstract]) OR (Paxil [Title/Abstract]) OR (Paroxetine Hydrochloride Hemihydrate [Title/Abstract]) OR (Paroxetine Hydrochloride, Hemihydrate [Title/Abstract]) OR (Paroxetine Hydrochloride [Title/Abstract])

#27 Search “Fluoxetine”[Mesh]

#28 Search (Fluoxetine [Title/Abstract]) OR (Fluoxetin [Title/Abstract]) OR (N-Methyl-gamma-(4-(trifluoromethyl)phenoxy)benzenepropanamine [Title/Abstract]) OR (Lilly-110140 [Title/Abstract]) OR (Lilly 110140 [Title/Abstract]) OR (Lilly110140 [Title/Abstract]) OR (Sarafem [Title/Abstract]) OR (Fluoxetine Hydrochloride [Title/Abstract]) OR (Prozac [Title/Abstract])

#29 Search “Fluvoxamine”[Mesh]

#30 Search (Fluvoxamine [Title/Abstract]) OR (Fluvoxadura [Title/Abstract]) OR (Fluvoxamin AL [Title/Abstract]) OR (Fluvoxamin beta [Title/Abstract]) OR (Fluvoxamin Stada [Title/Abstract]) OR (Fluvoxamin-neuraxpharm [Title/Abstract]) OR (Fluvoxamin neuraxpharm [Title/Abstract]) OR (Fluvoxamin-ratiopharm [Title/Abstract]) OR (Fluvoxamin ratiopharm [Title/Abstract]) OR (ratio-Fluvoxamine [Title/Abstract]) OR (ratio Fluvoxamine [Title/Abstract]) OR (Fluvoxamina Geminis [Title/Abstract]) OR (Geminis, Fluvoxamina [Title/Abstract]) OR (Fluvoxamine Maleate [Title/Abstract]) OR (Fluvoxamine Maleate, (E)-Isomer [Title/Abstract]) OR (Fluvoxamine, (Z)-Isomer [Title/Abstract]) OR (Luvox [Title/Abstract]) OR (Floxyfral [Title/Abstract]) OR (Fevarin [Title/Abstract]) OR (Dumirox [Title/Abstract]) OR (Faverin [Title/Abstract]) OR (Novo-Fluvoxamine [Title/Abstract]) OR (Novo Fluvoxamine [Title/Abstract]) OR (Nu-Fluvoxamine [Title/Abstract]) OR (Nu Fluvoxamine [Title/Abstract]) OR (PMS-Fluvoxamine [Title/Abstract]) OR (PMS Fluvoxamine [Title/Abstract]) OR (Desiflu [Title/Abstract]) OR (DU-23000 [Title/Abstract]) OR (DU 23000 [Title/Abstract]) OR (DU23000 [Title/Abstract])

#31 Search “Sertraline”[Mesh]

#32 Search (Sertraline [Title/Abstract]) OR (Zoloft [Title/Abstract]) OR (Altruline [Title/Abstract]) OR (Lustral [Title/Abstract]) OR (Apo-Sertraline [Title/Abstract]) OR (Apo Sertraline [Title/Abstract]) OR (Aremis [Title/Abstract]) OR (Besitran [Title/Abstract]) OR (Sealdin [Title/Abstract]) OR (Gladem [Title/Abstract]) OR (Novo-Sertraline [Title/Abstract]) OR (Novo Sertraline [Title/Abstract]) OR (ratio-Sertraline [Title/Abstract]) OR (ratio Sertraline [Title/Abstract]) OR (Rhoxal-sertraline [Title/Abstract]) OR (Rhoxal sertraline [Title/Abstract]) OR (Sertraline Hydrochloride [Title/Abstract]) OR (Hydrochloride, Sertraline [Title/Abstract]) OR (Sertraline Hydrochloride (1S-cis)-Isomer [Title/Abstract]) OR (Gen-Sertraline [Title/Abstract]) OR (Gen Sertraline [Title/Abstract])

#33 Search “Amoxapine”[Mesh]

#34 Search (Amoxapine [Title/Abstract]) OR (2-Chloro-11-(1-piperazinyl)dibenz(b,f)(1,4)oxazepine [Title/Abstract]) OR (Desmethylloxapine [Title/Abstract]) OR (CL-67,772 [Title/Abstract]) OR (CL 67,772 [Title/Abstract]) OR (CL67,772 [Title/Abstract]) OR (Demolox [Title/Abstract]) OR (Asendin [Title/Abstract]) OR (Défanyl [Title/Abstract]) OR (Asendis [Title/Abstract])

#35 Search “Amitriptyline”[Mesh]

#36 Search (Amitriptyline [Title/Abstract]) OR (Tryptine [Title/Abstract]) OR (Amineurin [Title/Abstract]) OR (Amitrip [Title/Abstract]) OR (Amitriptylin Beta [Title/Abstract]) OR (Amitriptylin Desitin [Title/Abstract]) OR (Desitin, Amitriptylin [Title/Abstract]) OR (Amitriptylin RPh [Title/Abstract]) OR (RPh, Amitriptylin [Title/Abstract]) OR (Amitriptylin-Neuraxpharm [Title/Abstract]) OR (Amitriptylin Neuraxpharm [Title/Abstract]) OR (Amitriptyline Hydrochloride [Title/Abstract]) OR (Amitrol [Title/Abstract]) OR (Anapsique [Title/Abstract]) OR (Apo-Amitriptyline [Title/Abstract]) OR (Apo Amitriptyline [Title/Abstract]) OR (Damilen [Title/Abstract]) OR (Domical [Title/Abstract]) OR (Laroxyl [Title/Abstract]) OR (Lentizol [Title/Abstract]) OR (Novoprotect [Title/Abstract]) OR (Saroten [Title/Abstract]) OR (Sarotex [Title/Abstract]) OR (Syneudon [Title/Abstract]) OR (Triptafen [Title/Abstract]) OR (Endep [Title/Abstract]) OR (Tryptizol [Title/Abstract]) OR (Elavil [Title/Abstract]) OR (Tryptanol [Title/Abstract])

#37 Search “Maprotiline”[Mesh]

#38 Search (Maprotiline [Title/Abstract]) OR (Maprotilin [Title/Abstract]) OR (N-Methyl-9,10-ethanoanthracene-9(10H)-propylamine [Title/Abstract]) OR (Dibencycladine [Title/Abstract]) OR (Psymion [Title/Abstract]) OR (Ludiomil [Title/Abstract]) OR (Maprolu [Title/Abstract]) OR (Maprotilin Holsten [Title/Abstract]) OR (maprotilin von ct [Title/Abstract]) OR (Maprotilin-neuraxpharm [Title/Abstract]) OR (Maprotilin neuraxpharm [Title/Abstract]) OR (Maprotilin-ratiopharm [Title/Abstract]) OR (Maprotilin ratiopharm [Title/Abstract]) OR (Maprotilin-TEVA [Title/Abstract]) OR (Maprotilin TEVA [Title/Abstract]) OR (Maprotiline Hydrochloride [Title/Abstract]) OR (Hydrochloride, Maprotiline [Title/Abstract]) OR (Maprotiline Mesylate [Title/Abstract]) OR (Mesylate, Maprotiline [Title/Abstract]) OR (Mirpan [Title/Abstract]) OR (Novo-Maprotiline [Title/Abstract]) OR (Novo Maprotiline [Title/Abstract]) OR (Ba-34,276 [Title/Abstract]) OR (Ba34,276 [Title/Abstract]) OR (Deprilept [Title/Abstract])

#39 Search “Nortriptyline”[Mesh]

#40 Search (Nortriptyline [Title/Abstract]) OR (Desitriptyline [Title/Abstract]) OR (Desmethylamitriptylin [Title/Abstract]) OR (Allegron [Title/Abstract]) OR (Apo-Nortriptyline [Title/Abstract]) OR (Apo Nortriptyline [Title/Abstract]) OR (Aventyl [Title/Abstract]) OR (Gen-Nortriptyline [Title/Abstract]) OR (Gen Nortriptyline [Title/Abstract]) OR (Paxtibi [Title/Abstract]) OR (Nortrilen [Title/Abstract]) OR (Nortriptyline Hydrochloride [Title/Abstract]) OR (Hydrochloride, Nortriptyline [Title/Abstract]) OR (Novo-Nortriptyline [Title/Abstract]) OR (Novo Nortriptyline [Title/Abstract]) OR (Nu-Nortriptyline [Title/Abstract]) OR (Nu Nortriptyline [Title/Abstract]) OR (Pamelor [Title/Abstract]) OR (PMS-Nortriptyline [Title/Abstract]) OR (PMS Nortriptyline [Title/Abstract]) OR (ratio-Nortriptyline [Title/Abstract]) OR (ratio Nortriptyline [Title/Abstract]) OR (Norfenazin [Title/Abstract])

#41 Search “Desipramine”[Mesh]

#42 Search (Desipramine [Title/Abstract]) OR (Desmethylimipramine [Title/Abstract]) OR (Demethylimipramine [Title/Abstract]) OR (Desipramine Hydrochloride [Title/Abstract]) OR (Hydrochloride, Desipramine [Title/Abstract]) OR (Norpramin [Title/Abstract]) OR (Ratio-Desipramine [Title/Abstract]) OR (Ratio Desipramine [Title/Abstract]) OR (Nu-Desipramine [Title/Abstract]) OR (Nu Desipramine [Title/Abstract]) OR (Pertofrane [Title/Abstract]) OR (Pertrofran [Title/Abstract]) OR (Pertofran [Title/Abstract]) OR (Petylyl [Title/Abstract]) OR (PMS-Desipramine [Title/Abstract]) OR (PMS Desipramine [Title/Abstract]) OR (Apo-Desipramine [Title/Abstract]) OR (Apo Desipramine [Title/Abstract]) OR (Novo-Desipramine [Title/Abstract]) OR (Novo Desipramine [Title/Abstract])

#43 Search “Trimipramine”[Mesh]

#44 Search (Trimipramine [Title/Abstract]) OR (Trimeprimine [Title/Abstract]) OR (10,11 Dihydro-N,N,beta-trimethyl-5H-dibenz(b,f)azepine-5-propanamine [Title/Abstract]) OR (Trimipramine, (-)-Isomer [Title/Abstract]) OR (Herphonal [Title/Abstract]) OR (Trimineurin [Title/Abstract]) OR (Novo-Tripramine [Title/Abstract]) OR (Novo Tripramine [Title/Abstract]) OR (NovoTripramine [Title/Abstract]) OR (Nu-Trimipramine [Title/Abstract]) OR (Nu Trimipramine [Title/Abstract]) OR (NuTrimipramine [Title/Abstract]) OR (Rhotrimine [Title/Abstract]) OR (Stangyl [Title/Abstract]) OR (Surmontil [Title/Abstract]) OR (Surmontil Maleate [Title/Abstract]) OR (Trimidura [Title/Abstract]) OR (Trimineurin Maleate [Title/Abstract]) OR (Trimipramin AZU [Title/Abstract]) OR (Trimipramin Beta [Title/Abstract]) OR (Beta, Trimipramin [Title/Abstract]) OR (Trimipramin Stada [Title/Abstract]) OR (Trimipramin-Neurazpharm [Title/Abstract]) OR (Trimipramin Neurazpharm [Title/Abstract]) OR (TrimipraminNeurazpharm [Title/Abstract]) OR (Trimipramine Maleate [Title/Abstract]) OR (Trimipramine Maleate (1:1) [Title/Abstract]) OR (Trimipramine Maleate (1:1), (+)-Isomer [Title/Abstract]) OR (Trimipramine Maleate (1:1), (+-)-Isomer [Title/Abstract]) OR (Trimipramine Maleate (1:1), (-)-Isomer [Title/Abstract]) OR (Trimipramine Mesylate [Title/Abstract]) OR (Trimipramine Monohydrochloride [Title/Abstract]) OR (Trimipramine, (+-)-Isomer [Title/Abstract]) OR (Eldoral [Title/Abstract]) OR (Apo-Trimip [Title/Abstract]) OR (Apo Trimip [Title/Abstract]) OR (ApoTrimip [Title/Abstract])

#45 Search “Imipramine”[Mesh]

#46 Search (Imipramine [Title/Abstract]) OR (Norchlorimipramine [Title/Abstract]) OR (Imidobenzyle [Title/Abstract]) OR (Imizin [Title/Abstract]) OR (Tofranil [Title/Abstract]) OR (Janimine [Title/Abstract]) OR (Melipramine [Title/Abstract]) OR (Pryleugan [Title/Abstract]) OR (Imipramine Pamoate [Title/Abstract]) OR (4,4'-Methylenebis(3-hydroxy-2-naphthoic acid)-3-(10,11-dihydro-5H-dibenzo(b,f)azepin-5-yl)-N,N-dimethyl-1-propanamine (1:2) [Title/Abstract]) OR (Imipramine Hydrochloride [Title/Abstract]) OR (Imipramine Monohydrochloride [Title/Abstract])

#47 Search “Protriptyline”[Mesh]

#48 Search (Protriptyline [Title/Abstract]) OR (Vivactil [Title/Abstract]) OR (Protriptyline Hydrochloride [Title/Abstract]) OR (Hydrochloride, Protriptyline [Title/Abstract])

#49 Search “Doxepin”[Mesh]

#50 Search (Doxepin [Title/Abstract]) OR (Deptran [Title/Abstract]) OR (Desidox [Title/Abstract]) OR (Doneurin [Title/Abstract]) OR (Doxepia [Title/Abstract]) OR (Doxepin beta [Title/Abstract]) OR (Doxepin Hydrochloride [Title/Abstract]) OR (Hydrochloride, Doxepin [Title/Abstract]) OR (Doxepin Hydrochloride, Cis-Trans Isomer Mixture (approximately 1:5) [Title/Abstract]) OR (Doxepin-RPh [Title/Abstract]) OR (Doxepin RPh [Title/Abstract]) OR (Espadox [Title/Abstract]) OR (Mareen [Title/Abstract]) OR (Novo-Doxepin [Title/Abstract]) OR (Novo Doxepin [Title/Abstract]) OR (Prudoxin [Title/Abstract]) OR (Quitaxon [Title/Abstract]) OR (Sinequan [Title/Abstract]) OR (Sinquan [Title/Abstract]) OR (Zonalon [Title/Abstract]) OR (Xepin [Title/Abstract]) OR (Aponal [Title/Abstract]) OR (Apo-Doxepin [Title/Abstract]) OR (Apo Doxepin [Title/Abstract]) OR (ApoDoxepin [Title/Abstract])

#51 Search “Clomipramine”[Mesh]

#52 Search (Clomipramine [Title/Abstract]) OR (Chlomipramine [Title/Abstract]) OR (Chlorimipramine [Title/Abstract]) OR (Hydiphen [Title/Abstract]) OR (Clomipramine Maleate (1:1) [Title/Abstract]) OR (Clomipramine Monohydrochloride [Title/Abstract]) OR (Monohydrochloride, Clomipramine [Title/Abstract]) OR (Anafranil [Title/Abstract]) OR (Clomipramine Hydrochloride [Title/Abstract]) OR (Hydrochloride, Clomipramine [Title/Abstract])

#53 Search “Bupropion”[Mesh]

#54 Search (Bupropion [Title/Abstract]) OR (Amfebutamone [Title/Abstract]) OR ((+-)-1-(3-Chlorophenyl)-2-((1,1-dimethylethyl)amino)-1-propanone [Title/Abstract]) OR (Wellbutrin [Title/Abstract]) OR (Zyban (Anti-Smoking) [Title/Abstract]) OR (Zyban (Bupropion) [Title/Abstract]) OR (Bupropion, (+-)-Isomer [Title/Abstract]) OR (Bupropion Hydrochloride [Title/Abstract]) OR (Bupropion Hydrochloride, (+-)-Isomer [Title/Abstract]) OR (Quomen [Title/Abstract]) OR (Zyntabac [Title/Abstract])

#55 Search “Mirtazapine”[Mesh]

#56 Search (Mirtazapine [Title/Abstract]) OR (6-Azamianserin [Title/Abstract]) OR (6 Azamianserin [Title/Abstract]) OR (ORG 3770 [Title/Abstract]) OR (ORG-3770 [Title/Abstract]) OR (ORG3770 [Title/Abstract]) OR (Rexer [Title/Abstract]) OR (Remeron [Title/Abstract]) OR (Norset [Title/Abstract]) OR (Remergil [Title/Abstract]) OR (Zispin [Title/Abstract]) OR (Esmirtazapine [Title/Abstract]) OR ((S)-Mirtazapine [Title/Abstract]) OR (Org 50081 [Title/Abstract]) OR ((N-Methyl-11C)mirtazapine [Title/Abstract])

#57 Search “Trazodone”[Mesh]

#58 Search (Trazodone [Title/Abstract]) OR (Tradozone [Title/Abstract]) OR (AF-1161 [Title/Abstract]) OR (AF 1161 [Title/Abstract]) OR (AF1161 [Title/Abstract]) OR (Deprax [Title/Abstract]) OR (Desyrel [Title/Abstract]) OR (Gen-Trazodone [Title/Abstract]) OR (Gen Trazodone [Title/Abstract]) OR (Molipaxin [Title/Abstract]) OR (Novo-Trazodone [Title/Abstract]) OR (Novo Trazodone [Title/Abstract]) OR (Trittico [Title/Abstract]) OR (PMS-Trazodone [Title/Abstract]) OR (PMS Trazodone [Title/Abstract]) OR (Ratio-Trazodone [Title/Abstract]) OR (Ratio Trazodone [Title/Abstract]) OR (RatioTrazodone [Title/Abstract]) OR (Thombran [Title/Abstract]) OR (Trazodon Hexal [Title/Abstract]) OR (Trazodon-Neuraxpharm [Title/Abstract]) OR (Trazodon Neuraxpharm [Title/Abstract]) OR (TrazodonNeuraxpharm [Title/Abstract]) OR (Trazodone Hydrochloride [Title/Abstract]) OR (Trazon [Title/Abstract]) OR (Apo-Trazodone [Title/Abstract]) OR (Apo Trazodone [Title/Abstract]) OR (Nu-Trazodone [Title/Abstract]) OR (Nu Trazodone [Title/Abstract])

#59 Search “agomelatine”[Supplementary Concept]

#60 Search (agomelatine [Title/Abstract]) OR (N-(2-(7-methoxy-1-naphthyl)ethyl)acetamide [Title/Abstract]) OR (AGO 178 [Title/Abstract]) OR (AGO178 [Title/Abstract]) OR (AGO-178 [Title/Abstract]) OR (Thymanax [Title/Abstract]) OR (Valdoxan [Title/Abstract]) OR (S20098 [Title/Abstract]) OR (S 20098 [Title/Abstract]) OR (S-20098 [Title/Abstract])

#61 Search “Reboxetine” [Mesh]

#62 Search (Reboxetine [Title/Abstract]) OR (Reboxetine Mesylate [Title/Abstract]) OR (2-((2-Ethoxyphenoxy)benzyl)morpholine methanesulfonate [Title/Abstract]) OR (Vestra [Title/Abstract])

#63 Search “Moclobemide” [Mesh]

#64 Search (Moclobemide [Title/Abstract]) OR (Moclobamide [Title/Abstract]) OR (Apo-Moclobemide [Title/Abstract]) OR (Apo Moclobemide [Title/Abstract]) OR (Arima [Title/Abstract]) OR (Aurorix [Title/Abstract]) OR (Manerix [Title/Abstract]) OR (Moclamine [Title/Abstract]) OR (Aurorex [Title/Abstract]) OR (Chem mart Moclobemide [Title/Abstract]) OR (Moclobemide, Chem mart [Title/Abstract]) OR (DBL Moclobemide [Title/Abstract]) OR (Moclobemide, DBL [Title/Abstract]) OR (Deprenorm [Title/Abstract]) OR (Feraken [Title/Abstract]) OR (GenRX Moclobemide [Title/Abstract]) OR (Moclobemide, GenRX [Title/Abstract]) OR (Healthsense Moclobemide [Title/Abstract]) OR (Moclobemide, Healthsense [Title/Abstract]) OR (Moclix [Title/Abstract]) OR (Terry White Chemists Moclobemide [Title/Abstract]) OR (Moclobemid Stada [Title/Abstract]) OR (Stada, Moclobemid [Title/Abstract]) OR (Moclobemid von ct [Title/Abstract]) OR (von ct, Moclobemid [Title/Abstract]) OR (Moclobemid-1A Pharma [Title/Abstract]) OR (Moclobemid 1A Pharma [Title/Abstract]) OR (Moclobemid1A Pharma [Title/Abstract]) OR (Moclobemid-Puren [Title/Abstract]) OR (Moclobemid Puren [Title/Abstract]) OR (Moclobemid-ratiopharm [Title/Abstract]) OR (Moclobemid ratiopharm [Title/Abstract]) OR (Moclobeta [Title/Abstract]) OR (Moclodura [Title/Abstract]) OR (Moclonorm [Title/Abstract]) OR (Nu-Moclobemide [Title/Abstract]) OR (Nu Moclobemide [Title/Abstract]) OR (NuMoclobemide [Title/Abstract]) OR (PMS-Moclobemide [Title/Abstract]) OR (PMS Moclobemide [Title/Abstract]) OR (Rimoc [Title/Abstract]) OR (Ro 11-1163 [Title/Abstract]) OR (Ro 11 1163 [Title/Abstract]) OR (Ro-11-1163 [Title/Abstract]) OR (Novo-Moclobemide [Title/Abstract]) OR (Novo Moclobemide [Title/Abstract]) OR (NovoMoclobemide [Title/Abstract]) OR (Moclobemid AZU [Title/Abstract]) OR (AZU, Moclobemid [Title/Abstract])

#65 Search “Selegiline” [Mesh]

#66 Search (Selegiline [Title/Abstract]) OR (Selegyline [Title/Abstract]) OR (Selegiline, (R)-Isomer [Title/Abstract]) OR (L-Deprenyl [Title/Abstract]) OR (Emsam [Title/Abstract]) OR (Selegiline Hydrochloride, (R,S)-Isomer [Title/Abstract]) OR (Selegiline Hydrochloride, (S)-Isomer [Title/Abstract]) OR (Eldepryl [Title/Abstract]) OR (Selegiline, (S)-Isomer [Title/Abstract]) OR (Yumex [Title/Abstract]) OR (Jumex [Title/Abstract]) OR (Humex [Title/Abstract]) OR (Zelapar [Title/Abstract]) OR (Deprenyl [Title/Abstract]) OR (E-250 [Title/Abstract]) OR (E 250 [Title/Abstract]) OR (E250 [Title/Abstract]) OR (Selegiline Hydrochloride, (R)-Isomer [Title/Abstract]) OR (Selegiline Hydrochloride [Title/Abstract]) OR (Selegiline, (R,S)-Isomer [Title/Abstract]) OR (Deprenalin [Title/Abstract]) OR (Deprenil [Title/Abstract])

#67 Search “Tianeptine” [Supplementary Concept]

#68 Search (Tianeptine [Title/Abstract]) OR ((3-chloro-6-methyl-5,5-dioxo-6,11-dihydrodibenzo(c,f)(1,2)thiazepin-11-yl)-7-aminoheptanoic acid [Title/Abstract]) OR (coaxil [Title/Abstract]) OR (tianeptine, monosodium salt [Title/Abstract]) OR (tianeptine, monosodium salt, (+-)-isomer [Title/Abstract]) OR (Stablon [Title/Abstract]) OR (tianeptine, (+-)-isomer [Title/Abstract]) OR

#69 Search “nefazodone” [Supplementary Concept]

#70 Search (nefazodone [Title/Abstract]) OR (Serzone [Title/Abstract]) OR (Dutonin [Title/Abstract]) OR (Nefadar [Title/Abstract]) OR (nefazodone hydrochloride [Title/Abstract]) OR (Lin-Nefazodone [Title/Abstract]) OR (Menfazona [Title/Abstract]) OR (Rulivan [Title/Abstract]) OR (Apo-Nefazodone [Title/Abstract])

#71 Search “Mianserin” [Mesh]

#72 Search (Mianserin [Title/Abstract]) OR (Mianserin Hydrochloride [Title/Abstract]) OR (Hydrochloride, Mianserin [Title/Abstract]) OR (Mianserin Monohydrochloride [Title/Abstract]) OR (Monohydrochloride, Mianserin [Title/Abstract]) OR (Tolvon [Title/Abstract]) OR (Lerivon [Title/Abstract]) OR (Org GB 94 [Title/Abstract])

#73 Search “Venlafaxine Hydrochloride” [Mesh]

#74 Search (Venlafaxine Hydrochloride [Title/Abstract]) OR (Hydrochloride, Venlafaxine [Title/Abstract]) OR (Cyclohexanol, 1-(2-(dimethylamino)-1-(4-methoxyphenyl)ethyl)-, hydrochloride [Title/Abstract]) OR (1-(2-(dimethylamino)-1-(4-methoxyphenyl)ethyl)cyclohexanol HCl [Title/Abstract]) OR (Wy 45030 [Title/Abstract]) OR (Wy-45030 [Title/Abstract]) OR (Wy45030 [Title/Abstract]) OR (Wy-45,030 [Title/Abstract]) OR (Wy 45,030 [Title/Abstract]) OR (Wy45,030 [Title/Abstract]) OR (Sila-Venlafaxine [Title/Abstract]) OR (Sila Venlafaxine [Title/Abstract]) OR (Effexor [Title/Abstract]) OR (Trevilor [Title/Abstract]) OR (Vandral [Title/Abstract]) OR (Efexor [Title/Abstract]) OR (Venlafaxine [Title/Abstract]) OR (Dobupal [Title/Abstract])

#75 Search “Duloxetine Hydrochloride” [Mesh]

#76 Search (Duloxetine Hydrochloride [Title/Abstract]) OR (Hydrochloride, Duloxetine [Title/Abstract]) OR (Duloxetine HCl [Title/Abstract]) OR (HCl, Duloxetine [Title/Abstract]) OR (LY 248686 [Title/Abstract]) OR (LY-248686 [Title/Abstract]) OR (LY248686 [Title/Abstract]) OR (Duloxetine Ethanedioate (1:1), (+-)-isomer - T353987 [Title/Abstract]) OR (LY 227942 [Title/Abstract]) OR (LY-227942 [Title/Abstract]) OR (LY227942 [Title/Abstract]) OR (Duloxetine [Title/Abstract]) OR (N-methyl-3-(1-naphthalenyloxy)-3-(2-thiophene)propanamide [Title/Abstract]) OR (N-methyl-3-(1-naphthalenyloxy)-2-thiophenepropanamine [Title/Abstract]) OR (Duloxetine, (+)-isomer [Title/Abstract]) OR (Cymbalta [Title/Abstract])

#77 Search “Phenelzine” [Mesh]

#78 Search (Phenelzine [Title/Abstract]) OR (beta-Phenylethylhydrazine [Title/Abstract]) OR (beta Phenylethylhydrazine [Title/Abstract]) OR (Fenelzin [Title/Abstract]) OR (Phenethylhydrazine [Title/Abstract]) OR (2-Phenethylhydrazine [Title/Abstract]) OR (2 Phenethylhydrazine [Title/Abstract]) OR (Phenelzine Sulfate [Title/Abstract]) OR (Sulfate, Phenelzine [Title/Abstract]) OR (Nardelzine [Title/Abstract]) OR (Nardil [Title/Abstract])

#79 Search “Tranylcypromine” [Mesh]

#80 Search (Tranylcypromine [Title/Abstract]) OR (trans-2-Phenylcyclopropylamine [Title/Abstract]) OR (trans 2 Phenylcyclopropylamine [Title/Abstract]) OR (Tranylcypromine Sulfate [Title/Abstract]) OR (Sulfate, Tranylcypromine [Title/Abstract]) OR (Jatrosom [Title/Abstract]) OR (Transamine [Title/Abstract]) OR (Parnate [Title/Abstract])

#81 #1 OR #2 OR #3 OR #4 OR #5 OR #6 OR #7 OR #8 OR #9 OR #10

#82 #11 OR #12

#83 #13 OR #14 OR #15 OR #16 OR #17 OR #18 OR #19 OR #20 OR #21 OR #22 OR #23 OR #24 OR #25 OR #26 OR #27 OR #28 OR #29 OR #30 OR #31 OR #32 OR #33 OR #34 OR #35 OR #36 OR #37 OR #38 OR #39 OR #40 OR #41 OR #42 OR #43 OR #44 OR #45 OR #46 OR #47 OR #48 OR #49 OR #50 OR #51 OR #52 OR #53 OR #54 OR #55 OR #56 OR #57 OR #58 OR #59 OR #60 OR #61 OR #62 OR #63 OR #64 OR #65 OR #66 OR #67 OR #68 OR #69 OR #70 OR #71 OR #72 OR #73 OR #74 OR #75 OR #76 OR #77 OR #78 OR #79 OR # 80

#84 #81 AND #82 AND #83
