# Supplementary material for: Comparative efficacy and safety of antidepressant therapy for the agitation of dementia: A systematic review and network meta-analysis
Source: Front Aging Neurosci. 2023 Mar 3;15:1103039. doi: 10.3389/fnagi.2023.1103039 (PMC10020338; doi:10.3389/fnagi.2023.1103039)
Supplement: Supplementary file 2 [file Data_Sheet_2.docx]

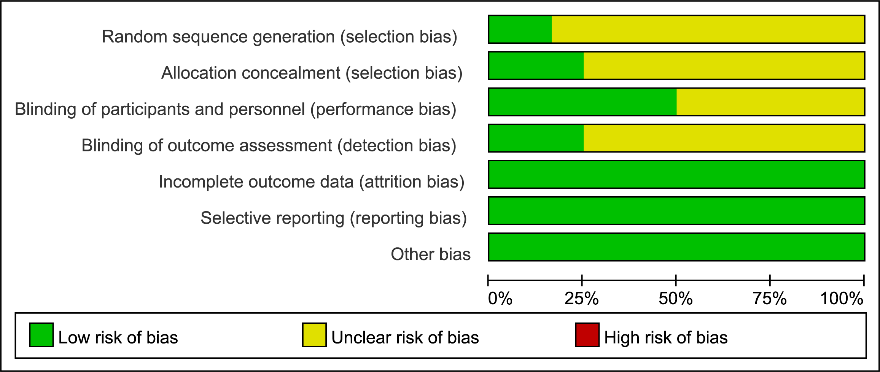

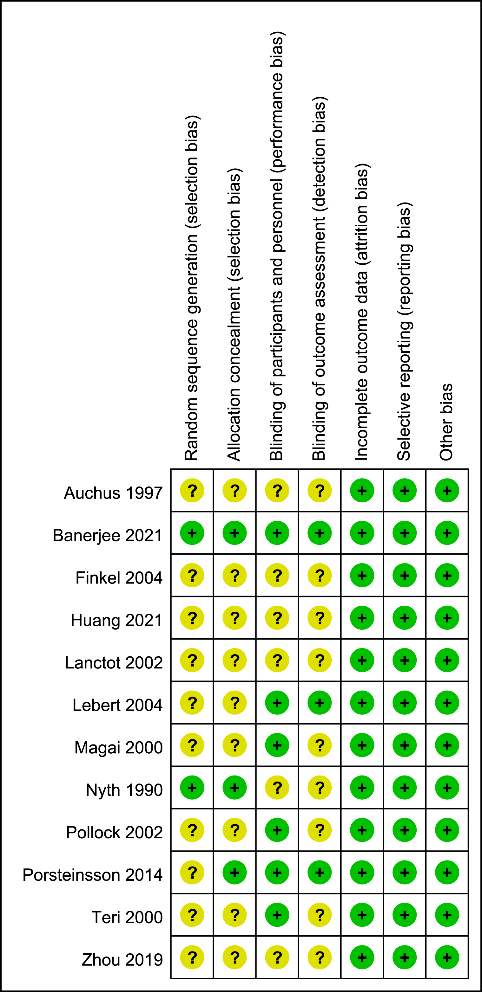


Appendix 2 Quality assessment for the bias risk of trials.


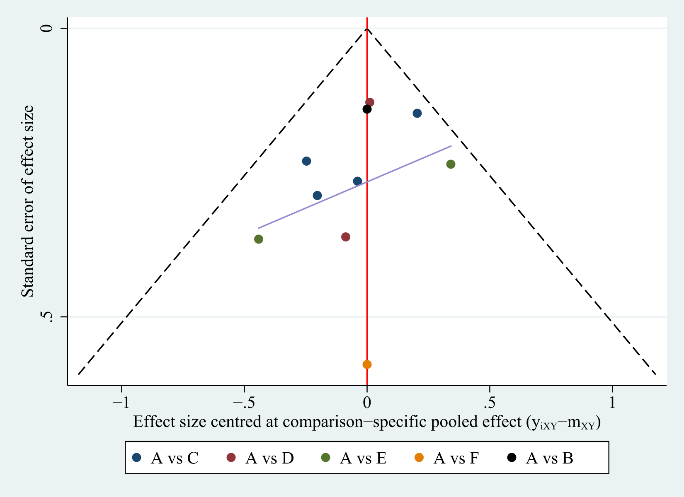

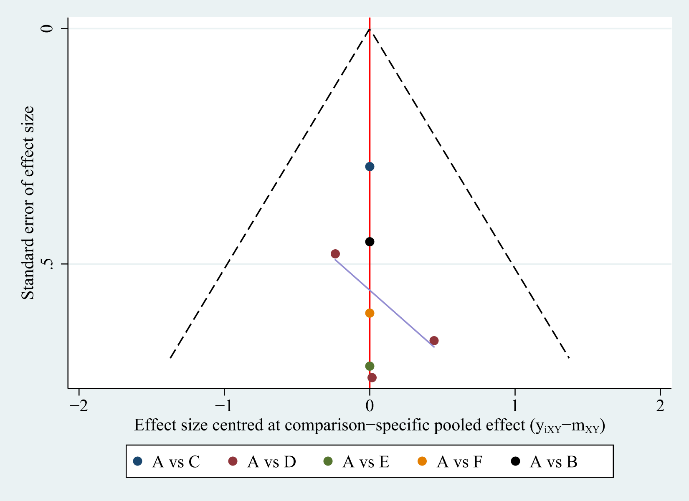


Appendix 3A The funnel plot of publication bias in the efficacy of the antidepressants.

A: Placebo

B: Mirtazapine

C: Citalopram

D: Sertraline

E: Trazodone

F: Fluoxetine

Appendix 3B The funnel plot of publication bias in the safety of the antidepressants.

A: Placebo

B: Escitalopram

C: Mirtazapine

D: Citalopram

E: Trazodone

F: Sertraline
